# Long-term fracture load of all-ceramic crowns: Effects of veneering ceramic thickness, application techniques, and cooling protocol

**DOI:** 10.4317/jced.57352

**Published:** 2020-11-01

**Authors:** Júlia-Magalhães-da Costa Lima, João-Paulo-Mendes Tribst, Lilian-Costa Anami, Renata-Marques de Melo, Dayanne-Monielle-Duarte Moura, Rodrigo-Othávio-Assunção Souza, Marco-Antonio Bottino

**Affiliations:** 1 DDs, MSc, PhD , University Hospital Lauro Wanderley, Federal University of Paraíba (UFPB), João Pessoa, PB, Brazil; 2 DDs, MSc, PhD, Department of Dental Materials and Prosthodontics, São Paulo State University (Unesp), Institute of Science and Technology, São José dos Campos, São Paulo, Brazil; 3 DDs, MSc, PhD, Departmentof Dentistry, Santo Amaro University, São Paulo, SP, Brazil; 4 DDs, MSc, PhD, Department of Dentistry, Division of Prosthodontics, Federal University of Rio Grande do Norte (UFRN), Natal/RN, Brazil

## Abstract

**Background:**

To evaluate, *in vitro*, the effects of the cooling protocol, application technique, and veneering ceramic thickness on the fracture resistance of ceramic crowns with Y-TZP frameworks.

**Material and Methods:**

80 frameworks were made from zirconia by the CAD/CAM technique and divided into 8 groups (n = 10) according to the factors: “application technique” (stratified-L and pressed -P), “thickness” (1 mm and 2 mm), and “cooling protocol” (slow-S and fast-F) of the feldspathic veneering ceramic. After, all crowns were cemented over G10 preparations with resin cement (Panavia F, Kuraray), mechanically cycled (2x106 cycles, 200 N, 3Hz), and subjected to the axial compression resistance test (0.5 mm/min, 10 kN). The data (N) underwent descriptive statistical analysis by 3-way ANOVA and Tukey’s test (5%). Fracture analysis was performed to determine the possible origin of failure.

**Results:**

The factors “cooling protocol” (*P*=0.0058) and “application” technique (*P*=0.0001) influenced the fracture resistance of the crowns. For pressed veneer technique, the P2S (4608.9±464.5). A presented significantly higher results than that P2F(3621.1±523.0)BCD (Tukey’s test). For the stratified technique, this difference was not observed (*P*>0.05). The thickness of the veneering ceramic was not significant regardless of the cooling protocol and technique (*P*>0.05). The predominant failure mode was chipping of the ceramic veneer originating in the subsurface.

**Conclusions:**

The pressed technique, used with a slow-cooling protocol, leads to the best outcome for the veneering of all-ceramic crowns.

** Key words:**Zirconia, ceramics, cooling protocol, thickness, application technique.

## Introduction

Several studies have been performed to understand the causes of the flaws found in all-ceramic restorations (1-4). The failure incidence of all-ceramic restorations is related to fractures in the ceramic veneer in 2% until 25% of the cases after 3 years of clinical use (5,6). In addition, this type of failure is more predominant than in metal-ceramic restorations, where 19.4% of failures were observed after 3 years of use (6,7). Several factors may be related to the fracture of all-ceramic restorations, as: material strength, fracture toughness, glass transition temperature, occlusal loads (8,9); anatomical support (9,10); shape and thickness of the framework and the veneering ceramics (9,11,12); ceramic application technique (3); and residual thermal stress (1-8).

Residual thermal stress is present in the ceramic material due to the temperature gradient and the difference between the coefficient of thermal expansion (CTE) between core and ceramic veneer. The residual stress is a relevant factor, widely reported as the major cause of fractures of the veneering ceramics in restorations with zirconia frameworks (13,14). In this context, it has been reported that the manufacture all-ceramic restorations involves a series of sintering thermal cycles and cooling processes, when each new layer of the veneering ceramics material is applied. According to Swain (1), during these firing cycles, the difference in CTEs among the materials (framework/veneering), and the cooling rate after each firing cycle can influence the magnitude of residual stress in the restoration.

When the CTE of the veneering ceramic is higher than that of the framework ceramic, tensile residual stress is generated in the veneering surface, leading to the development of the chipping type of failure. However, if the CTE of the framework is higher than the veneering ceramic, delamination can occur detaching the aesthetic ceramic and the core, due to the tensile residual stresses generated at the interface (1,4). The cooling rate can generate compressive residual stresses in the veneering ceramic surface and tensile residual stresses in the subsurface. There is a directly proportional relationship among cooling rate, thickness, and residual stress development: smaller thickness and slower cooling will lead to lower residual stress in the veneering ceramic (1).

However, it is important to note that the cooling rate – and hence the amount of residual stress – depends on the thickness and the geometry of the specimen (15). In this way, a slow-cooling protocol should be established and tested in Y-TZP prostheses covered by ceramic with clinically relevant geometry (16). Another important factor is the manufacturing technique that can be associated with the cohesive failure of the veneering ceramic observed in clinics (16). The layering technique is more sensitive, due to the consecutive applied layers of veneering ceramic, the repetitive sintering firings (17), and the possibility of voids and defects incorporation (18).

Thus, this study aimed to evaluate the effects of thickness, application technique, and the cooling rate of the veneering ceramic on the fracture resistance of all-ceramic crowns with zirconia framework. The hypothesis tested was that the application technique of veneering ceramic, as well as the cooling protocol and the thickness, would influence the fracture resistance of all-ceramic crowns.

## Material and Methods

-Sample preparation

An anatomical preparation corresponding to a human first molar (6 mm high and ending in a 1.2 mm chamfer) was designed with three-dimensional (3D) modeling software (Rhinoceros 4.0, Seattle, WA, USA). From the 3D design, 80 preparations were milled in an epoxy-resin-based material reinforced by glass fiber NEMA grade G-10 (International Paper, Hampton, SC, USA), which has mechanical properties similar to those of human dentin (19).

Each G10 preparation had its base embedded in acrylic resin, and one of them was scanned (inEos Blue, Sirona Dental Systems, Bensheim, Germany). A 3D image was generated with the scanner software (Sirona Dental Systems) the finishing line of the preparation was demarcated, the insertion axis was determined and the software virtually designed the crown’s framework. From this design, 80 identical zirconia frameworks were obtained from pre-sintered blocks of tetragonal zirconia partially stabilized by yttrium oxide (VITA In-Ceram YZ for inLab, Vita Zahnfabrik) and were milled in a CAD/CAM facility (InLab CEREC MC XL, Sirona Dental Systems).

Before sintering, frameworks were cleaned with distilled water in an ultrasound bath for 5 min, then were subjected to a cleaning firing (VITA Vacumat 6000 MP, Vita Zahnfabrik) and were immersed in (Coloring Liquid, LL1, Vita Zahnfabrik) for two minutes, according to one of the protocols recommended by the manufacturer. The sintering process was performed as recommended by the manufacturer (rising time, 1.5 h; end temperature, 1530 °C; holding time for end temperature, 2 h; cooling to 400 °C with firing chamber kept closed). After sintering, the zirconia frameworks and G10 preparations were randomly distributed into 8 groups (n = 10) according to the factors: “application technique”, “thickness”, and “cooling protocol of the veneering ceramic” (Table 1). A calibrated technician applied the veneering ceramic by two different techniques according to the groups (Table 2).

**Table 1:**
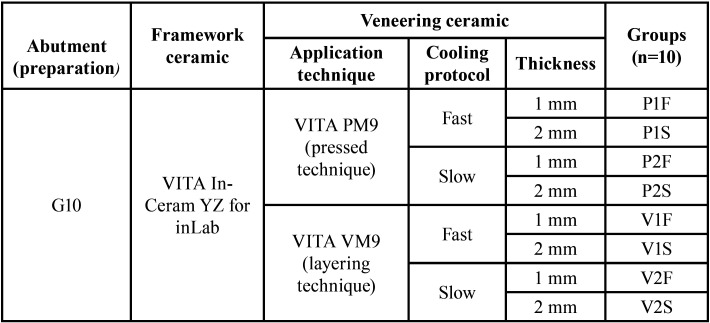
Experimental design according to the different application technique and cooling protocol.

**Table 2:**
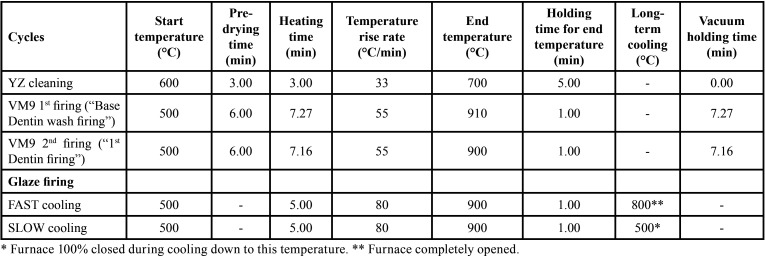
Sintering cycles for each ceramic material.

-Heat-pressed Technique

For standardization of the veneer ceramic design, polymer blocks (VITA CAD Waxx, Vita Zahnfabrik) were milled (InLab CEREC MC XL, Sirona Dental Systems) in two different thickness (1 and 2 mm in the major groove of the occlusal surface). Twenty crowns of each thickness were obtained.

The polymer crowns were placed over the zirconia framework, and a small amount of sculpture wax was applied to the crown to seal the margin and fix the structures. The sprues were attached to the crowns, and the set was positioned at the center of the sprue-former at a 45° angle. The coating (Bellavest HS Bego, Bremen, Germany) was mixed and then poured into the silicone mold according to the manufacturer’s instructions. After setting, the coating ring was placed in a preheated oven (850 °C) for at least 75 minutes for evaporation of the wax and the polymer.

The ring was immediately transferred to the ceramic oven, and two VITA PM9 Tablets (2M1P – Opaque, Vita Zahnfabrik) were pressed. The cycle was performed according to the manufacturer’s instructions: firing began at 700 °C with 0 min of pre-drying and 6 min of temperature rise at 50 °C per/min for up to 1000 °C, which was maintained for 20 min, and 3 bar of pressure for 8 min.

To remove the crowns, the coating was cut with cutting discs mounted in a hand motor and then sandblasted with 50-µm particles of aluminum oxide (Asfer Chemical Ltda, São Caetano do Sul/SP, Brazil).

-Layering Technique

From the two types of crowns obtained by the heat-pressed technique, two molds of silicone were made (Elite HD, Zetaplus System, Zhermack, Badia Polesine, Italy) to standardize all crowns in the present study.

The zirconia frameworks were cleaned in an ultrasound bath with distilled water for 5 min, and the veneering ceramic (VITA VM9, Base Dentine 1M1, Vita Zahnfabrik) was applied by the layering technique with the aid of the molds. The framework surfaces were coated with a mixture of ceramic powder and the modeling liquid (VITA VM9, Modeling Liquid, Vita Zahnfabrik), by means of a brush, and condensed by manual vibration. The excess water was removed with absorbent tissues, and sintering (Table 2) was performed. Two dentin firings were performed to compensate the shrinkage of approximately 12% that occurred after the firing cycles.

Forty bilayer crowns were made with the same dimensions as the heat-pressed ones: 20, 1 mm thick and 20, 2 mm thick in the major groove of the occlusal surface.

-Cooling protocols

A layer of glaze (VITA Akzent, Vita Zahnfabrik) was applied on the crowns, and after the respective firing cycle (Table 2), the crowns were subjected to two different cooling procedures.

During fast cooling, the ceramic furnace (VITA Vacumat 6000 MP Vita Zahnfabrik) was programmed to open immediately after the firing cycle (Table 2), and the crowns were removed from the base of the oven to cool at room temperature (25°C) (20).

For slow cooling, the same furnace was used (Table 2) and was programmed to remain tightly closed during cooling until the temperature of 500°C was reached. Then, the oven was opened, and the crowns were kept in the base of the oven until it reached room temperature. This protocol was based on a previous report (20).

-Cementation procedure

All crowns were cemented to the preparations with a dual-cured resin cement (Panavia F. Kuraray Medical Inc.).

The G10 preparations were etched with 5% hydrofluoric acid gel for 60 sec. The acid was removed with air/water spray for 30 sec and ultrasonically cleaned with distilled water for 5 min. The preparations were dried with oil-free air jets for 30 sec, and the silane agent (Clearfil SE Bond Primer and Porcelain Bond Activator, Kuraray Medical Inc.) was applied with a disposable brush. After 5 sec, a gentle air jet was applied, and the silane was allowed to evaporate for 60 sec. The ED Primer (Kuraray Medical Inc.) was applied to the preparations. After 60 sec, it was gently air-dried.

Equal amounts of the resinous cement were mixed for 20 sec, and applied to the inner margins of the zirconia framework. Each crown was initially positioned with slight manual pressure over the preparation, and then a 750 g load was applied at the occlusal surface. The excess cement was removed, and four 40-second polymerizations were carried out around the set (0°, 90°, 180°, 270°) with a curing light (Curing Light Radii-Cal, SDI, Bayswater, VIC, Australia). The cemented samples were immersed in distilled water at 37 °C for 24 hours before the mechanical cycling.

-Mechanical cycling 

All crowns were subjected to 2x106 cycles of 200 N at 3Hz in a mechanical cycling machine (Erios 11000, Erios Technical and Scientific Equipment Ltd., São Paulo, Brazil) immersed in distilled water at 37°C (21). The load applicators consisted of solid stainless steel spherical tips (6 mm in diameter) positioned in the center of the occlusal surface of each crown. An acetate strip was interposed between load applicators and crowns.

After mechanical cycling, each crown was examined in a stereomicroscope (30x, Discovery V20, Zeiss, Jena, Germany) to confirm the absence of chipping or failures due to cycling.

A compressive test was conducted in a universal testing machine (EMIC DL 1000, São José dos *Pi*nhais/PR, Brazil), at a speed of 0.5 mm/min and a 10 kN load cell with a load applicator similar to that used in mechanical cycling. During the test, the samples were kept immersed in distilled water at 37 °C with the aid of a thermostat, to simulate conditions similar to those of the oral environment. Compression resistance was recorded in N for each crown at the first sign of failure.

-Failure Analysis

The specimens were examined by stereomicroscopy (70x, Discovery V2, Zeiss) to determine the failure (22). The classification based on the type of fracture: crack - cracking of the veneering ceramic at the interface; chipping - fracture on the surface of the veneering ceramic without exposure of the framework; delamination - fracture of the veneering ceramic with exposure of the framework; and catastrophic - fracture of the veneering ceramic and zirconia framework (23,24). The representative fragments were analyzed by scanning electron microscopy (Inspect S50, FEI Company, Brno, Czech Republic) (9,16).

-Statistical Analysis

The sample’s power was calculated through the website www.openepi.com, considering a 95% confidence interval. After checking the assumptions for normality and homogeneity, the fracture load data were subjected to inferential statistical analysis by parametric variance (3-way ANOVA) and multiple comparison by Tukey’s test (α = 0.05), (Table 3).

**Table 3:**
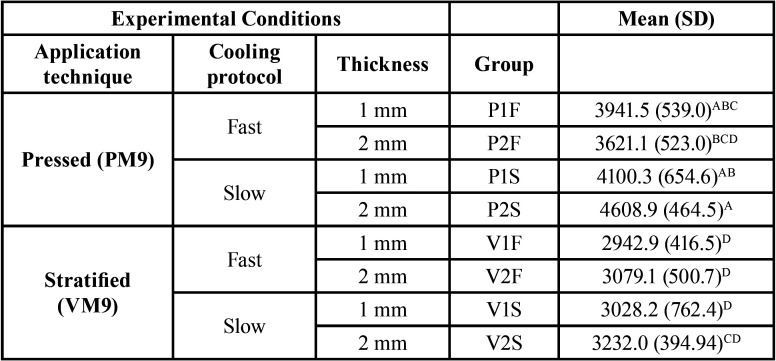
Mean (SD) values of the fracture load (in N) for the experimental groups and Tukey’s grouping.

## Results

The Power for fracture load data was 96%. The mean and standard deviation (SD) of the maximum force of fracture load (N) and the comparison among experimental groups (Tukey’s test 5%) are presented in Table 4. ANOVA showed that the cooling protocol (F=8.08; *P*= 0.0058) and application technique (F=67.1; *P*= 0.0001) were statistically significant. For pressed veneer technique, the P2S (4608.9±464.5)A presented significantly higher results than that P2F(3621.1±523.0)BCD. For the stratified technique, no difference among groups was observed (*P*>0.05).

**Table 4:**
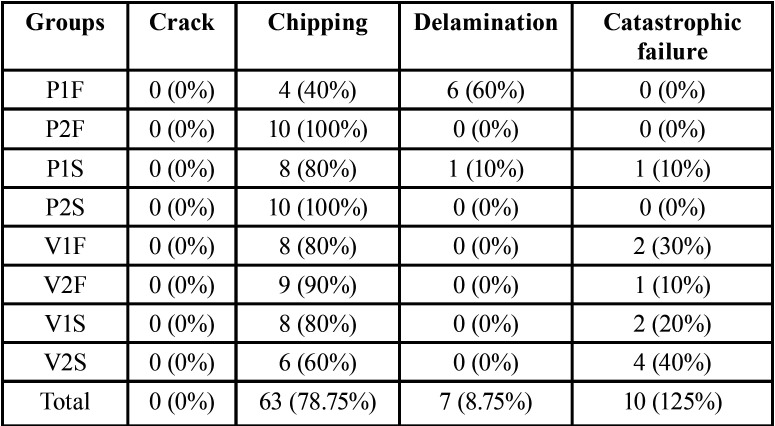
Data distribution according to failures mode classification for each experimental group: number of samples and percentage.

**Figure 1:**
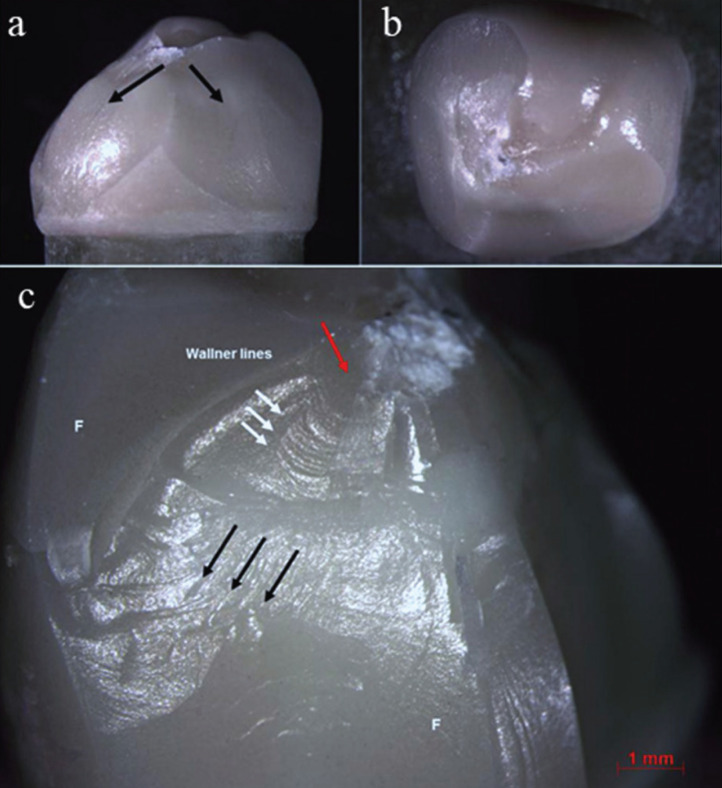
a) Representative stereomicroscope image (7,5x) of a cone-shaped fracture propagation (cone crack) of the veneering ceramic, b) chipping without framework failure of a crown from group V1F, c) Stereomicroscope image (10x) of a P2S crown failed by chipping. The black arrows indicate the direction of the failure in the feldspathic ceramic; the red arrow indicate the area of the probable failure’s origin and the white arrow indicate the wallner lines. F: feldspathic veneering ceramic.

**Figure 2:**
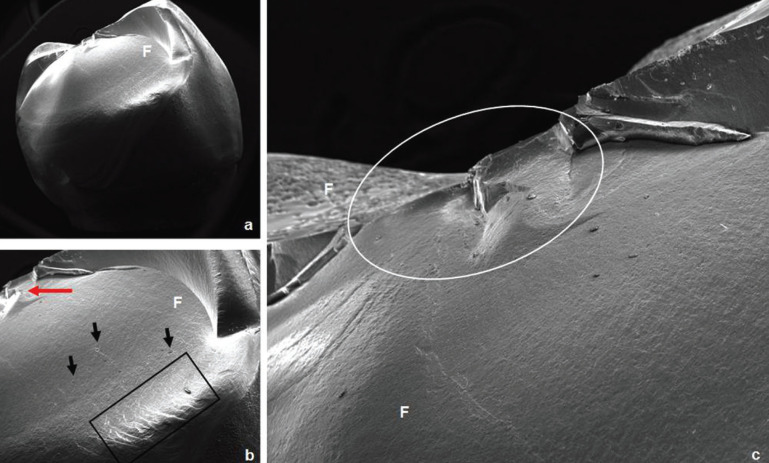
SEM photomicrographs of a crown from P2S group failed by chipping. a) Overview of the fracture (24x). b) The red arrow indicates the area of load application; the presence of wake hackles (black arrows) indicates the direction of fracture propagation; it is possible to observe the compression curl (black rectangle) (70x). c) Closer image of the area under the load application (white ellipse); it can be seen that the pressed technique shows fewer porosities (90x). F: feldspathic veneering ceramic.

**Figure 3:**
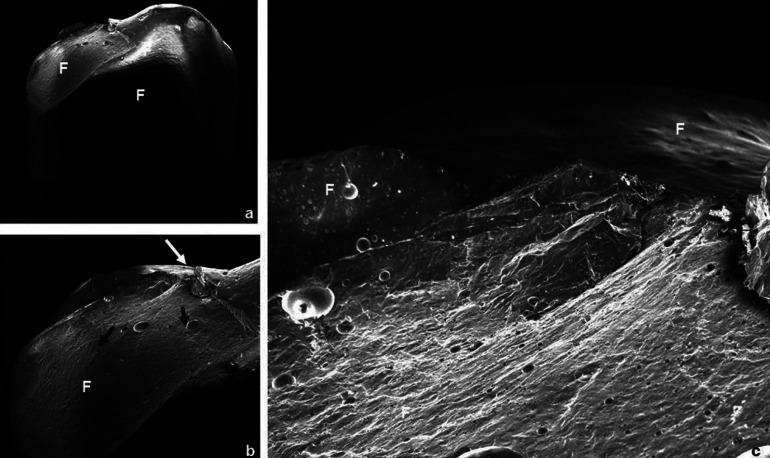
SEM photomicrographs of a crown from V2F group failed by chipping. a) Overview of the fracture (22x). b) The area of load application is indicated by the white arrow; the presence of wake hackles (black arrows) indicates the direction of failure propagation (60x). c) Closer view of the area under the load application; it can be observed the presence of many bubbles resulted from the stratified technique (150x). F: feldspathic veneering ceramic.

The failures analysis showed that all crowns presented cone-shaped fracture propagation (cone-crack) of the veneering ceramic from the point of load application, as illustrated in Figure 1. Fractographic analysis showed that the cone-crack began in the subsurface, a few microns below the outer surface where the load was applied (Figs. 2,3). The failure mode classifications are shown in Table 4.

## Discussion

This study was designed to evaluate the effect of thickness, application technique, and the cooling rate of the veneering ceramic on the fracture resistance of all-ceramic crowns with zirconia framework. For this, to simulate the influence of aging (25) on the fracture load of all-ceramic crowns, the mechanical cycling was performed with 2 million cycles and a 200 N load, simulating a longer period (ca. 8.5 years) of clinical use (26,27). The applied load simulate the chewing in the posterior area (246.9 to 2091.9 N with an average of (776.7 N) (28).

In Roark’s Formulas (29), the solution to measure the contact pressure of a ‘sphere on sphere’ case was used to calculate the stress between the cusp and the sphere in the present study. The highest load-to-fracture (N) of the present study and a cusp curvature radius of ~5,5.10-3 m to calculate the maximum contact pressure on the cusp during fracture, which was ~17.103 MPa. The maximum chewing force mentioned previously corresponds to ~13.103 MPa. This means that, in a real crown, the material would hardly break under the forces applied in the present study, although fatigue in the mouth tends to diminish the fracture load of materials.

Nevertheless, fracture can occur when an excessive load is applied to the crown, due to parafunction such as clenching and bruxism, in which the forces applied are of great magnitude and short duration (24,30-32).

The results of the present study were higher than those reported previously (23,33-35), especially for heat-pressed crowns cooled slowly. The reported diversity of values for fracture load may be due to the different designs of the specimens, thicknesses, resinous cement, and different materials used to simulate the tooth preparation (36).

Similar to other studies (4,8,34,36), this study considered crowns with clinically relevant anatomy, varying the thickness of the veneering ceramic and keeping their shapes similar, despite the application technique or the thickness of the veneering ceramic. Crowns with anatomical frameworks have higher fracture toughness and lower incidence of fractures in the veneering ceramic (5,36), often resulting in failures of easy repair and without functional and esthetic impairment (35). Comparing fracture strength of non-anatomical and anatomical crowns, a previous study (34) found that the negative effects of high brittleness of the veneering ceramics were reduced in anatomical crowns.

Furthermore, the fractures of the veneering ceramic were greater in extent for non-anatomical crowns (1). It can occurs because the adequate support for the veneering ceramic creates a favorable conditions to reduce the stress, increasing the fracture load. In addition, the greater thicknesses of the zirconia in non-anatomical crowns generate more residual tensile stress, resulting in higher incidence of fractures (34).

According to the present study results, the hypothesis that the application technique of veneering ceramic, as well as the cooling protocol and the thickness, would influence the fracture resistance of all-ceramic crowns, was partially accepted. Regarding the thickness, it has been reported that thicker veneering ceramic are related to reduced restoration strength, due to the ceramic is susceptibility to subsurface residual stresses (1,2,33). However, the restoration thickness depends on the antagonist tooth, occlusal space, preparation and anatomy (35). Some studies claim that greater thicknesses of veneering ceramic are related to lower load to fracture (1,2,33). Smaller ceramic thicknesses led to reduced stress generated due to shrinkage during the cooling process (11). It would thus be expected that resistance would be better with smaller thicknesses. However, in this study, thickness did not influence fracture resistance.

The veneering ceramic application technique is a factor that can influence the mechanical properties of the restorations when the inherent characteristics of the materials are considered (33,35). In the present study, the mechanical properties of the two veneering ceramics used are similar (PM9, 100 MPa, 9.0-9.5x10-6K-1; VM9, 100 MPa, 8.0-9.2x10-6K-1) (35) however statistically significant differences was found for the application technique. Therefore, it is believed that the technique is a significant factor regardless the restorative material properties. The lower fracture resistance values of the layering restoration can be explained by the increased sintering cycles in which the crown is subjected, resulting in a higher tensile residual stress, especially in areas without support (35), despite the fact that this is a more sensitive and critical technique (37,38). The best results found for the heat-pressed crowns can be explained by the higher density and reduced incorporation of failures (porosities), since this technique is more controlled than the layering technique (18). The heat-pressed technique showed the best results for both cooling protocols, especially for slow cooling.

A previous study that used anatomical crowns to compare the two application techniques found lower fracture load for heat-pressed technique. The authors believe that the lack of experience with the heat-pressed technique may have influenced the results (35). Moreover, these authors did not consider the influence of slow cooling on fracture load, which proved to be a significant factor in the present study.

Regarding the cooling protocol, the results of the present study showed that when the slow-cooling and the heat-pressed techniques were adopted, there was a significant increase in fracture load. During the manufacture of the crowns, whether fully ceramic or metal-ceramic, several ceramic layers are applied, to obtain the required anatomy and achieve the desired anatomy. Each layer is subjected to sintering cycles with temperatures significantly above the glass transition temperature of the veneering ceramic (usually around 600°C) (1,3). A slower cooling temperature is important in the final firing cycle, once the stress introduced in the first firing cycle is relaxed in the next firing cycle (1).

The predominant failure type was chipping of the veneering ceramic, and there were a few samples with the infrastructure impairment. These findings are consistent with those of clinical report that found a higher number of cohesive failures in which a quantity of veneering ceramic remained on the framework (34). An *in vitro* study (35) also observed cohesive failure as the predominant failure mode. In the present study, determination of the exact origin of failure was hampered due to the high load required for fracture of these crowns, resulting in areas of destruction under the region of load application. Some findings, such as wake hackles and Wallner lines, indicated the direction of crack propagation.

In conclusion, the fracture resistance of zirconia framework crowns is not influenced by veneering ceramic thickness. The pressed technique, used with a slow-cooling protocol, leads to the best outcome for the veneering of all-ceramic crowns.
